# *Liquid biopsy*: from discovery to clinical implementation

**DOI:** 10.1002/1878-0261.12997

**Published:** 2021-06-01

**Authors:** Catherine Alix‐Panabières, Klaus Pantel

**Affiliations:** ^1^ Laboratory of Rare Human Circulating Cells (LCCRH) University Medical Centre of Montpellier Montpellier France; ^2^ CREEC/CANECEV MIVEGEC (CREES) University of Montpellier CNRS IRD Montpellier France; ^3^ Department of Tumor Biology University Cancer Center Hamburg University Medical Center Hamburg‐Eppendorf Hamburg Germany

‘Liquid biopsy’ was introduced as a new diagnostic concept in 2010 [1] for the analysis of circulating tumor cells (CTCs) in the blood of cancer patients and has been now extended to the analysis of circulating tumor‐derived factors, in particular, cell‐free tumor DNA (ctDNA), as well as extracellular vesicles (EVs), cell‐free microRNAs (cfmiRNAs), mRNA, long noncoding RNA, small RNA, circulating cell‐free proteins, and tumor‐educated platelets (TEPs). Liquid biopsy is a minimally invasive procedure usually based on sampling of blood, but also of cerebrospinal fluid, urine, sputum, ascites, and theoretically any other body fluid [2].

Over the past decade, various methods have been developed to detect CTCs and ctDNA in the peripheral blood of cancer patients [3, 4]. While reliable information can be easily obtained in patients with advanced disease, early‐stage cancer patients usually present with very low concentrations of CTCs and ctDNA [5]. At present, most CTC assays rely on epithelial markers and the majority of CTCs detected are single isolated cells. The clinical relevance of ‘mesenchymal’ CTCs lacking any epithelial markers, as well as CTC clusters, is still under investigation. The biology of EpCAM and its role is not completely understood; however, evidence suggests that the expression of this epithelial cell‐surface protein is crucial for metastasis‐competent CTCs and may not be lost completely during the epithelial‐to‐mesenchymal transition [6, 7, 8]. Although most published studies have been performed on patients with carcinomas and melanomas, CTCs have been also detected in the peripheral blood of patients with primary brain tumors (glioblastomas) despite the blood–brain barrier [9].

Liquid biopsy assays are currently being validated for early detection of cancer, which is supposed to reduce cancer‐related mortality. Despite remarkable progress, liquid biopsy‐based detection of early stages of solid cancers remains a challenge. New blood‐based biomarkers for early detection currently validated in clinical trials include miRNAs, exosomes, and tumor‐educated platelets [10, 11, 12].

In patients with diagnosed cancer, CTCs and ctDNA analyses can obtain independent information on prognosis in early and advanced stages of disease. In particular, CTC counts at initial diagnosis are able to refine the current risk stratification by TNM staging in early‐stage breast cancer and have been therefore included into the 2018 AJCC classification. Moreover, early detection of relapse by sequential ctDNA (or CTCs) analysis of blood samples obtained post‐surgery during the follow‐up is possible and may be used in future trials to stratify patients to ‘post‐adjuvant’ therapies [13].

With the growing body of evidence concerning the prognostic value of CTCs, scientists and clinicians began to investigate the clinical utility of CTCs *via* interventional clinical trials. The STIC CTC METABREAST is the first study that has demonstrated the clinical utility of CTCs in assigning people with metastatic breast cancer to either chemotherapy or hormonal therapy [14]. In particular, patients with elevated CTC counts who were assigned to the low‐risk group by the conventional physician decision profited from chemotherapy.

Another key application of liquid biopsy is to identify therapeutic targets or mechanisms of resistance of metastatic cells in individual patients [3]. While the analysis of ctDNA focuses on mutations relevant for cancer therapy (including EGFR, KRAS, or ESR1 mutations), CTCs offer a wide spectrum of analyses at the DNA, RNA, and protein levels [5, 15]. Metastatic cells might have unique characteristics that can differ from the bulk of cancer cells in the primary tumor, currently used for stratification of patients to systemic therapy. Moreover, monitoring of CTCs and ctDNA before, during, and after systemic therapy (e.g., chemotherapy, hormonal therapy, antibody therapy) might provide unique information for the future clinical management of the individual cancer patient and might serve as a surrogate marker for response to therapy. In the context of recent success in antibody‐mediated blockade of immune checkpoint control molecules [16], the expression of PD‐L1 on CTCs might be of interest as potential predictive marker [17, 18]. Moreover, Lu *et al*. [19] reported that DNA sensing within tumor cells is essential for anti‐tumor immunity triggered by DNA mismatch repair deficiency (dMMR). This recent study provides new mechanisms and biomarkers for anti‐dMMR‐cancer immunotherapy. In addition, the expression of androgen receptor variant 7 in CTCs may predict resistance to anti‐androgen therapy in prostate cancer, while mutations in the estrogen receptor gene (ESR1) provide information on resistance to hormone therapy in breast cancer [4]. Additional therapeutic targets detected on CTCs in cancer patients include the estrogen receptor and HER‐2 oncogene [5]. Single‐cell RNAseq analysis of CTCs may provide more comprehensive information on relevant pathways [20, 21].

For functional analysis of CTCs, the development of *in vitro* and *in vivo* test systems has started, which might also serve as models for drug testing [22, 23, 24]. In particular, the development of cell lines and xenografts derived from CTCs can provide novel insights into the biology of tumor cell dissemination and may be used to discover new pathways to target specifically metastatic cells [25, 26].

Besides CTCs and ctDNA, the analysis of circulating microRNAs, exosomes, or tumor‐educated platelets may provide complementary information as ‘liquid biopsy’. Indicatively, the integrin composition of exosomes seems to determine the organ site of metastatic niches and the RNA expression pattern of blood platelets reveals information on tumors in cancer patients [27].

Sensitive methods have been also developed to capture disseminated tumor cells (DTCs) in the bone marrow in cancer patients [13], which provide new insights into the process of ‘cancer dormancy’. The nature of dormant breast cancer cells and the mechanisms leading to their outgrowth are poorly understood. Efforts to unravel the nature of cancer dormancy have been hampered by the lack of sensitive methods to detect dormant cells in cancer patients. Very recently, Albrengues *et al*. [28] found that lung inflammation (induced by either tobacco smoke exposure or nasal instillation of lipopolysaccharide) awakened dormant cancer cells and converted them to aggressive lung metastases in the mouse model. Currently, the potential correlation between inflammation or smoking, neutrophil extracellular traps (NETs), and recurrence after dormancy in human patients needs to be tested. If such a link can be established, NETs and their downstream effectors could be targeted to reduce the risk of cancer recurrence in human patients. The development of novel therapies designed to kill dormant residual tumor cells, or maintain them in a quiescent state, represents a highly attractive approach to prevent late recurrence. Such an approach, however, would require a far more detailed understanding of tumor dormancy and recurrence than exists today, as well as biomarkers to enable monitoring of this process and predict recurrence. Analysis of DTCs leads to the discovery of new molecules relevant to the biology of metastasis such as the putative metastasis‐suppressor RAI2 [29].

This special issue on current challenges of liquid biopsy research includes seven articles written by experts in this field of research and covers multiple facets of liquid biopsy:

- CTCs – Vasseur *et al*. [30] summarize the evidence that has established CTCs as an independent prognostic factor in several cancer types (clinical validity), in both localized and metastatic settings, with a particular focus on breast cancer, and the published or ongoing phase II‐III clinical trials designed to demonstrate the clinical utility of CTCs. Moreover, Labib *et al*. [31] review key technologies used to isolate and analyze CTCs and discuss recent clinical studies that examined CTCs for genomic and proteomic predictors of responsiveness to therapy. Finally, current limitations that still hamper the implementation of CTCs into clinical practice are pointed out.
- ctDNA – In this issue, Filipska and Rosell [32] focus on the detection and clinical relevance of ctDNA in lung cancer, which is a suitable model tumor type for druggable mutations and the use of immunotherapies. In particular, the detection of druggable mutations has opened new avenues to liquid biopsy for stratification and monitoring of targeted therapies and the determination of tumor mutational burden has been explored as predictive biomarker for immunotherapy in NSCLC.
- Epigenetic biomarkers in CTCs and ctDNA – Blood‐based epigenetic biomarkers have a high potential for early cancer detection, since tumor‐specific DNA methylation patterns in plasma are known to arise early during cancer pathogenesis. DNA methylation might be also influenced by the specific homing tissue of CTCs and might therefore provide information on tumor localization. Lianidou summarizes the latest findings on DNA methylation markers in ctDNA for early detection, prognosis, minimal residual disease, risk of relapse, treatment selection and resistance, for the four most frequent tumor entities (breast, prostate, lung, and colorectal cancer) [33].
- DNA content of EVs (EV‐DNA)–Although most EV studies have so far focused on RNA or protein content, various types of DNA have been associated with exosomes, including mitochondrial DNA and genomic DNA. Elzanowska *et al*. [34]. discuss the biology and clinical applications of EV‐DNA. In detail, they review EV‐DNA biogenesis and mechanisms of DNA loading into EVs, as well as the various functions of DNA‐carrying EVs. Importantly, they examine current implications for EV‐DNA in the clinical setting, specifically in cancer diagnosis.
- Protein biomarkers – Proteins found in blood plasma have a long history as biomarkers of cancer, one that predates the several more recently introduced classes of biomarkers for liquid biopsy. One known biomarker is prostate‐specific antigen (PSA), which is used in prostate cancer diagnostics, although it has been associated with frequent false positives. A slowly expanding range of other protein markers commonly used to detect malignancy includes AFP (alpha‐fetoprotein), CA‐125, CEA (carcinoembryonic antigen), CA15‐3, CA19‐9, and a few more, but their clinical utility is limited, even when they are applied in combination [2]. Landegren and Hammond comment on the poor molecular detection sensitivity of current protein assays compared to nucleic acid detection reactions and discuss requirements for achieving detection of vanishingly small amounts of proteins, to ensure detection of early stages of malignant growth through liquid biopsy [35].
- Tumor‐educated platelets – The capacity of platelets to take up proteins and nucleic acids and alter their megakaryocyte‐derived transcripts and proteins in response to external signals makes them an interesting liquid biopsy source. Even if platelets are routinely isolated through well‐established and fast methods in clinical diagnostics, their value as a source of cancer biomarkers has been only recently introduced.
  - Antunes‐Ferreira *et al*. [36] highlighted the fact that platelets are important repositories of potential cancer biomarkers, including several types of RNAs (mRNA, miRNA, circRNA, lncRNA, and mitochondrial RNA) and proteins. The authors listed the preclinical studies showing the potential of tumor‐educated platelets as a liquid biopsy source for detecting various types and stages of cancer and included a valuable discussion on lacking information for their implementation into clinical practice.

In conclusion, liquid biopsy analysis can be used to obtain new insights into metastasis biology, and as companion diagnostics to improve the stratification of therapies and to obtain insights into therapy‐induced selection of cancer cells. In this context, intra‐patient tumor heterogeneity may represent an important mechanism to escape the complete eradication of all tumor clones by targeted therapies [37]. Researchers and clinicians have known about the clinical potential of liquid biopsies for many years. To push them into widespread use, more interventional clinical trials are now needed, as well as the development of an algorithm to combine the appropriate circulating biomarkers [38]. The next generation of liquid biopsies is increasingly going toward the analysis of complex cancer liquid biopsy data and will require a greater role for machine learning and artificial intelligence [39]. Technical and clinical assay validation is very important and can be achieved in international consortia such as the European Liquid Biopsy Society (ELBS) network (www.elbs.eu).

## Conflict of interests

CA‐P received honoraria from Menarini; KP received honoraria from Menarini, Illumina, and Agena.
